# Learning to localise weakly-informative sound spectra with and without feedback

**DOI:** 10.1038/s41598-018-36422-z

**Published:** 2018-12-18

**Authors:** Bahram Zonooz, Elahe Arani, A. John Van Opstal

**Affiliations:** Biophysics Department, Donders Center for Neuroscience, Radboud University, Heyendaalseweg 135, 6525 AJ Nijmegen, The Netherlands

## Abstract

How the human auditory system learns to map complex pinna-induced spectral-shape cues onto veridical estimates of sound-source elevation in the median plane is still unclear. Earlier studies demonstrated considerable sound-localisation plasticity after applying pinna moulds, and to altered vision. Several factors may contribute to auditory spatial learning, like visual or motor feedback, or updated priors. We here induced perceptual learning for sounds with degraded spectral content, having weak, but consistent, elevation-dependent cues, as demonstrated by low-gain stimulus-response relations. During training, we provided visual feedback for only six targets in the midsagittal plane, to which listeners gradually improved their response accuracy. Interestingly, listeners’ performance also improved without visual feedback, albeit less strongly. Post-training results showed generalised improved response behaviour, also to non-trained locations and acoustic spectra, presented throughout the two-dimensional frontal hemifield. We argue that the auditory system learns to reweigh contributions from low-informative spectral bands to update its prior elevation estimates, and explain our results with a neuro-computational model.

## Introduction

**Figure 1:**
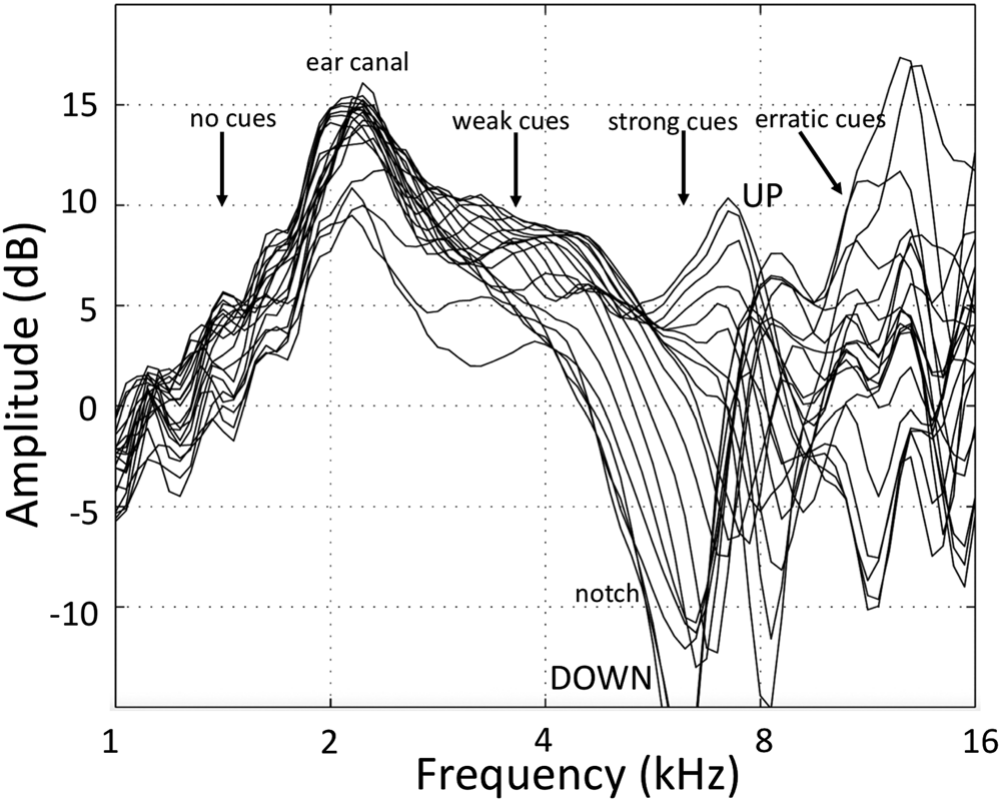
HRTFs (log-amplitude spectra as function of log-frequency) from a representative human subject, measured at the eardrum for different elevation angles (−50 < ε < 60 deg; downward and upward HRTFs are indicated). Note that although the strongest spectral cues are found in the notch region between 5–10 kHz, the different curves start to diverge as a systematic function of elevation already around 3–3.5 kHz (’weak cues’). The spectral peak near 2.5 kHz is caused by the first resonance in the ear canal at λ/4 ≈ 3.5 cm and is direction-independent. Above 10 kHz, the auditory system has poor sensitivity and the spectral cues become nonmonotonic and erratic.

Human sound localisation relies on the use of acoustic interaural difference cues in time (ITDs) and level (ILDs) for sources in the horizontal plane (azimuth angle, α), and of direction-dependent spectral shape cues, induced by the acoustic filtering of the head and pinnae, for locations in the medial plane (elevation: up-down and front-back angle, ε). The latter are described by direction-dependent Head Related Transfer Functions (HRTFs)^1–4^, and are illustrated in Fig. 1. It has been demonstrated that independent binaural and monaural neural pathways in the auditory brainstem process the different localisation cues^2,3,5–9^.

However, the auditory system has to overcome several fundamental problems in order to reliably use these cues for unambiguous and veridical source localisation. First, there is a set of locations, described by the so-called cone of confusion, for which the ITDs and ILDs are all identical^1,10^. For example, all locations in the midsagittal plane have ITD = 0 μs, and ILD = 0 dB. Thus, the ILDs and ITDs alone cannot uniquely specify the direction of a sound source, as they leave room for considerable spatial ambiguity.

The system could resolve this fundamental problem by estimating the target’s elevation angle from the spectral-shape cues of the pinnae^11,12^. However, here a second problem arises, since the sensory input at the eardrum, s(t; ε), is always determined by the linear convolution between the sound-source pressure wave, x(t), and the elevation-dependent impulse response function of the pinna filter, h(t;ε), both of which are a-priori unknown to the brain^3,13^. Hence, the estimation of source elevation is a fundamentally ill-posed problem (one measurement, two unknown quantities), which cannot be uniquely resolved either. Taken together, veridical sound localisation performance seems impossible to achieve.

Yet, despite these fundamental problems, open-loop free-field localisation experiments have indicated that the human auditory system can reliably localise and segregate a wide class of sound sources in the environment with high accuracy and precision^3,13,14^. It is therefore thought that the auditory system makes additional prior assumptions about sound sources and acoustic cues, to successfully cope with the ambiguity problems, and to enable a statistically (near-)optimal estimate of the sound-source direction, given the current sensory input^12,15^. Through a continuous evaluation of its stimulus-response behaviour in relation to the spectral-temporal sensory input, the brain could thus update its internal priors to maintain optimized localisation estimates.

Indeed, earlier studies have demonstrated considerable plasticity in human sound-localisation behaviour^16–18^, and effects of perceptual training that enhanced sound-localisation performance in adult ferrets^19^. For example, if the human pinnae are modified by small bilateral moulds, the acoustic spectral-shape patterns change, and sound localisation in the elevation direction is no longer possible. However, over the course of days to weeks, localisation abilities gradually improve, until reaching near-normal performance levels^16,20–22^. In case of a unilateral mould, localisation performance selectively adapts on the affected, ipsilateral side^18^. It is thought that several factors could contribute to this learning: visual feedback to identify localisation errors in daily life^17,23^, planning and making active orienting movements of eyes and head to sound sources^20,22,24,25^, changes in internal expectations through updating priors^26^, etcetera.

Sound localisation in the median plane requires broadband sound spectra that cover the relevant features of the pinna filters^13,15,27–29^. These filters contain their direction-dependent information for frequencies above about 4–5 kHz^30^. Typically, in humans, the most prominent directional cues are found in the 5–10 kHz band (the so-called notch region), but the surrounding spectral bands may also contain relevant directional information, albeit weaker, or less reliable^29,31^ (Fig. 1). So far, little is known about the underlying computational mechanisms of source-elevation estimation.

In the present study, we explored the mechanisms of perceptual learning in the human auditory system for stimuli with a limited bandwidth between 0.5–6 kHz, by providing visual feedback for a small number of source locations in the midsagittal plane during the training phase. All subjects improved their response accuracy to these sounds, which contained weak, but consistent, spatial cues. We tested sound-localisation performance, before and after the training, to different spectral stimuli at a large number of locations distributed across the two-dimensional frontal hemifield, to assess whether the learning had generalised beyond the training set. In a separate experiment, we trained the localization of poor spectral stimuli without providing any feedback. Again, listeners gradually expanded their response range and improved their accuracy. We explain our results with a neurocomputational model, in which the auditory system re-weighs the different spectral regions of the sensory input, before comparing the sensory spectrum to stored spatial-spectral information from its own pinna filters.

## Results

### Experiment 1: training with visual feedback

#### Controls

The control experiments to targets in the central frontal hemifield revealed that all participants were well able to localise broadband Gaussian White Noise (GWN) sounds. In addition, they all had considerable problems in localising the stimuli with degraded to poor spectral content (BS15, BS25, BS35, and LP6, respectively; see Fig. S1 in Supplemental Information). Nonetheless, the responses to these stimuli also demonstrated some spatial sensitivity for upward vs. downward locations, suggesting that their auditory systems were able to determine an elevation estimate on the basis of weak spectral information. Table 1 summarizes the average regression results (with standard deviations) for the response elevation components across subjects for all five stimuli of the control experiment. Note that the band-stop (BS) stimuli yielded much poorer localisation results (and more idiosyncratic variability) than the GWN stimuli, but gains, biases, and variability measures remained relatively high. Best localisation performance was obtained for the BS15 stimuli, for which the spectral notch region was attenuated the least. The low-pass (LP6) stimuli however, were consistently localised with the poorest spatial resolution, i.e., with the lowest response gain, and highest response bias. Still, even for these stimuli, the gains for their localisation performance differed significantly from zero (p < 1e-4), indicating a consistent positive contribution of the low-frequency spectral cues (the’weak cues’ in Fig. 1) to the elevation percept.

**Table 1:** Elevation results from the control experiment.

Stim	gain (std)	bias (std) (deg)	r^2^ (std)
GWN	0.87 ± 0.20	3 ± 8	0.78 ± 0.22
BS15	0.63 ± 0.21	1 ± 10	0.68 ± 0.22
BS25	0.49 ± 0.18	2 ± 10	0.59 ± 0.23
BS35	0.50 ± 0.14	1 ± 8	0.60 ± 0.21
LP6	0.35 ± 0.19	11 ± 9	0.40 ± 0.18

Regression results (gain, bias and coefficient of determination, *r*^*2*^. Values report means and standard deviations across all subjects) for the five different stimulus types used in the experiments: GWN, three different band-stop sounds in which the central notch region (6–9 kHz) was attenuated by 15 dB, 25 dB, and 35 dB, respectively, and low-pass filtered GWN with a high-frequency cut-off at 6 kHz.

#### Pre-training

**Figure 2:**
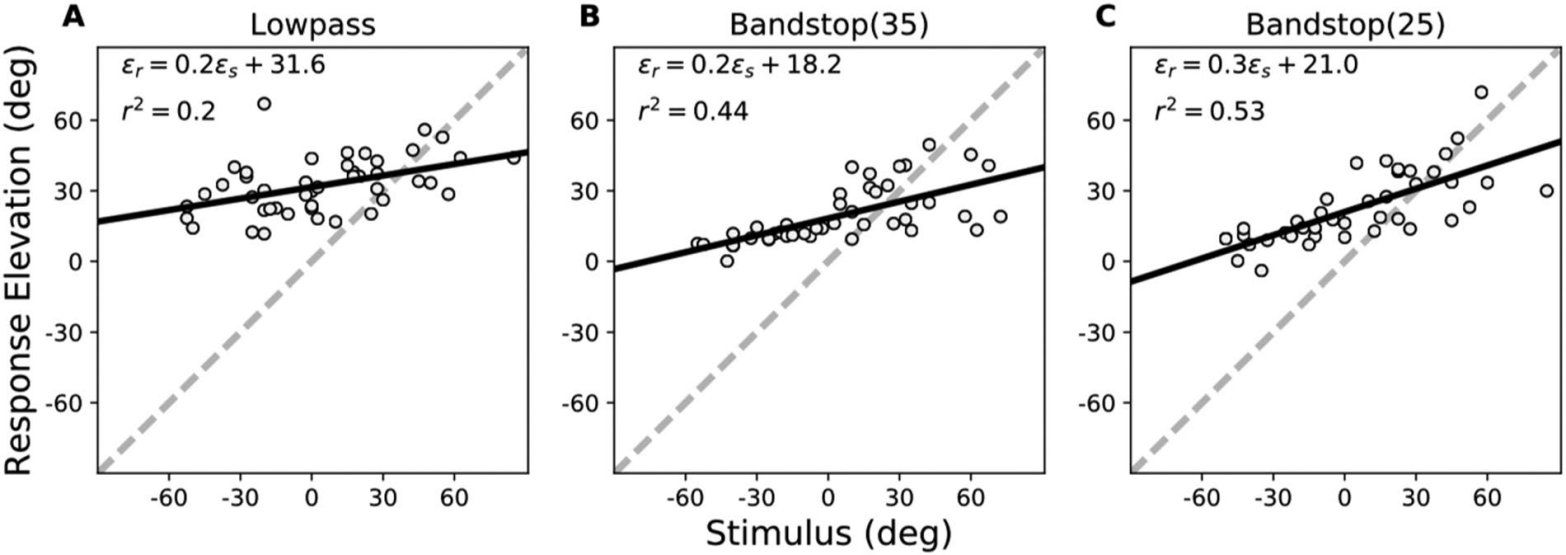
Pre-training results for subject S8 in elevation for the three test stimuli. Dashed diagonals: perfect response behaviour. Note that responses were highly inaccurate for all three stimuli, as gains and biases deviated substantially from their optimal values of 1.0 and 0.0 deg, respectively. Yet, the positive slopes of the three regression lines differed significantly from zero, indicating that the stimuli still contained some elevation-dependent spectral cues. Note also the differences in response variability, indicated by *r*^*2*^.

In the pre-training experiment, we measured localisation performance for three spectrally degraded stimuli (LP6, BS35, and BS25, see Fig. S1) presented across the two-dimensional frontal hemifield (Fig. S2). The results for the stimulus-response relationships of the elevation components for representative subject S8 are shown in Fig. 2). The localisation regression data indicate the precision and accuracy with which this listener responded to the sound sources. Like in the control experiments (Table 1), which covered a smaller azimuth-elevation range, localisation performance was severely degraded for all three stimuli (lower gain, higher bias and more variability than for the standard GWN stimuli), albeit in different ways. Especially the LP6 stimuli yielded a higher response bias (>30 deg) and a lower correlation coefficient, when compared to the BS25 and BS35 stimuli (bias ∼ 20 deg). In all three cases, however, the regression model yielded a significant sensitivity of this subject to changes in target elevation (p < 1e-3), demonstrating that the auditory system was able to map the weak spectral cues into a systematic elevation estimate.

#### Training

**Figure 3:**
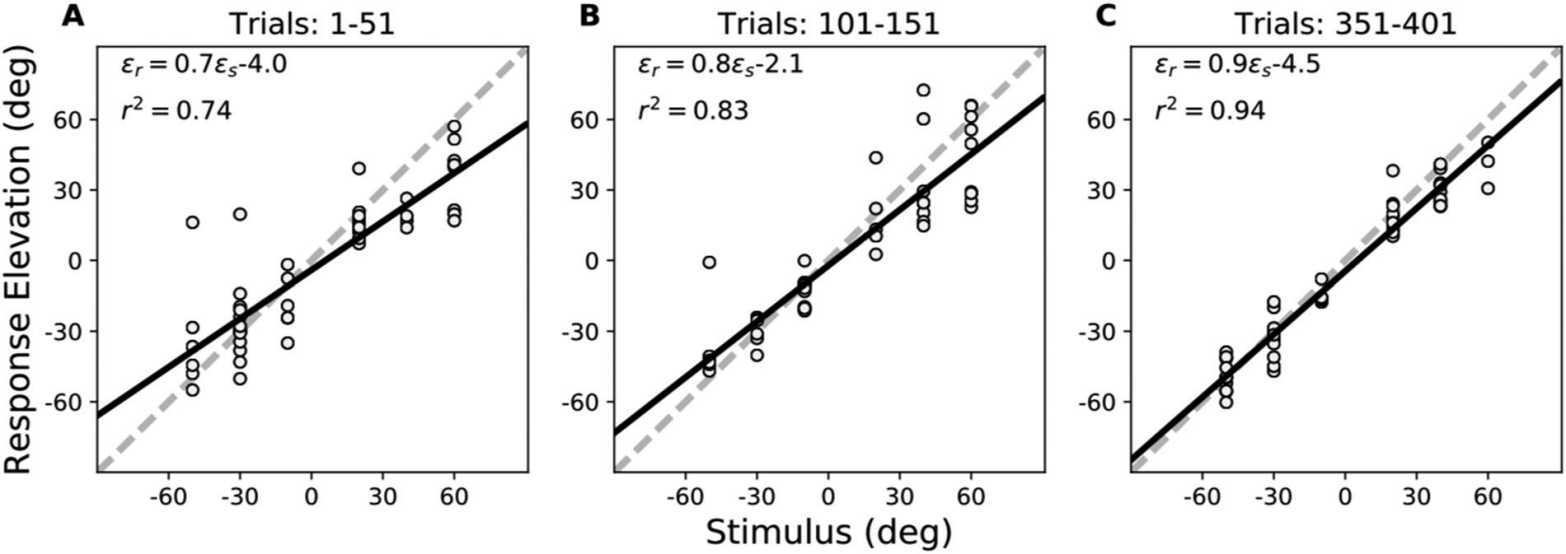
Localisation data for the six training targets in the midsagittal plane (BS35 stimuli) presented in randomized order and followed by visual feedback (Fig. S2) (**A**) at the start (trials 1–51), (**B**) after 100 training trials (nrs. 101–151), and (**C**) towards the end of the session (trials 351–401). Note the systematic increase of the response gain, and the reduction in response variability (increased *r*^*2*^) during the session. Data from subject S8.

To investigate whether explicit error feedback could improve the localisation accuracy of spectrally poor, yet weakly informative, sound sources in elevation, subjects were exposed to a training session of about 500 trials, in which they responded with head-orienting saccades to the six selected B35 stimulus locations (ε_T_ ∈ [−50,−30, −10, +20, +40, +60] deg) in the midsagittal plane (Fig. S2 in SI). About 1.5–2.5 sec later, the sound was followed by the presentation of an LED at the center of the target speaker, and the subject was required to make a correction head movement towards the LED, immediately after the sound-localisation response. Stimulus locations were selected in pseudo-randomised order. Figure 3 shows some representative sound-evoked response data for three subsequent 50-trial epochs during the training session: at the start of the training (trials 1–51), after the initial phase of the training (trials 101–151), and towards the end of the training (trials 351–401). Comparing the three epochs, it can be noted that response accuracy and precision both improved as the training progressed: the gain systematically increased from g_ε_ = 0.7 to g_ε_ = 0.9, while at the same time the response variability decreased, as the coefficient of determination improved from r^2^ = 0.74 to r^2^ = 0.94. As a result, both the residual errors and the mean absolute localisation error (MAE) for the six targets systematically decreased during the training session.

**Figure 4:**
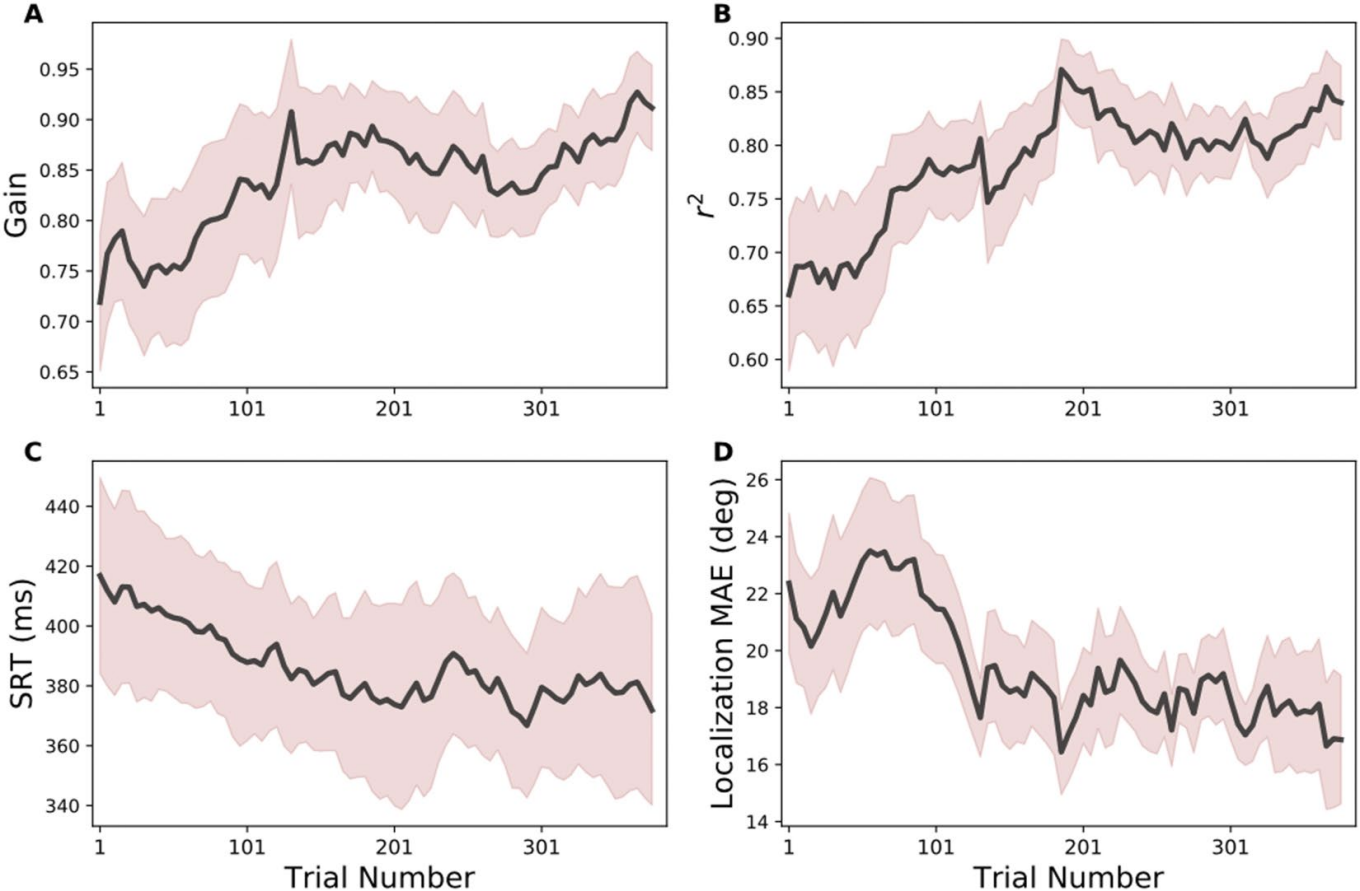
The influence of visual feedback on localisation performance to the six midsagittal target locations as function of time during the training session. (**A**) Elevation gain; (**B**) localisation precision (*r*^*2*^); (**C**) head-saccade reaction time; (**D**) mean absolute error. Values were determined by windowed regression, and averaged across subjects. The solid black line and shaded area indicate mean and standard deviation, respectively.

Similar results were obtained for the other eight subjects. To illustrate the subjects’ learning patterns during the overall time course of the training session, we performed a windowed regression analysis on the data of every subject (by taking a window of 50 subsequent trials per regression, and shifting the analysis across the session in steps of 5 trials), and averaged the results across subjects. The data (mean: solid line, and standard deviation: pink shading) are shown in Fig. 4. The elevation response gain (Fig. 4A), and the localisation precision (*r*^2^) (Fig. 4B) systematically increased with trial number, while the head-saccade reaction times (Fig. 4C) and the MAE (Fig. 4D) systematically decreased during the training. Note that all response measures seemed to reach their final performance levels around trial number 200. The co-variation of response variability with reaction time suggests that the auditory system becomes faster with increased confidence about the perceived source location.

#### Post-training

During the training, subjects had been exposed to a single stimulus type from which the 6–9 kHz band had been attenuated by 35 dB (BS35; Fig. S1B in SI). Moreover, sounds were presented from a very limited number of possible locations (n = 6, all confined to the midsagittal plane). Rather than true spectral-spatial learning, subjects could in principle have improved their response behaviour merely by categorizing or memorizing the six locations on the basis of subtle acoustic cues. For example, it is conceivable that the different speakers introduced minor speaker-specific colouring of the spectrum, leading to a particular chroma of the sound, which subjects might have learned to recognise during the session with visual feedback. If so, the improved response behaviour would persist only for the particular trained stimulus conditions, and not generalise across the two-dimensional frontal hemifield, where many other speakers were used, or to other spectrally degraded sounds with clearly different perceptual chroma.

**Figure 5:**
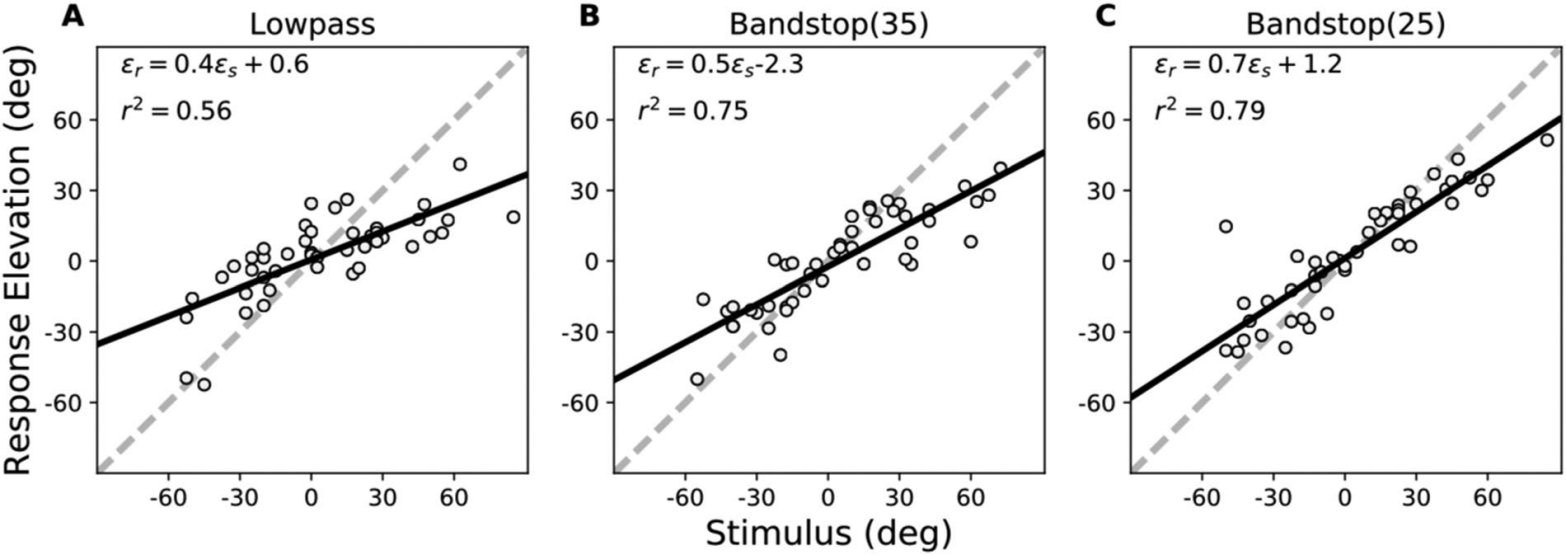
Post-training results for subject S8 in elevation for the same three test stimuli as in Fig. 2. Note the clear improvement in response accuracy and precision for all three stimuli, as gains and *r*^*2*^ values increased, and biases decreased substantially when compared to their pre- training values.

To establish whether the training had indeed resulted in improved sound-localisation performance across the entire frontal hemifield, as well as for different sound spectra, we re-tested the subjects after the training with the same three stimulus types and source locations as in the pre-training session. The regression analyses for the head-orienting responses of subject S8 for these three stimuli are shown in Fig. 5. The results show a clearly improved performance when compared with the data of Fig. 2. The response accuracy and precision for the BS35 stimuli had increased from *g*_*ε*_ = 0.2 and *r*^2^ = 0.44 for the pre-training phase, to post-training values of *g*_ε_ = 0.5 and *r*^2^ = 0.75, respectively. In addition, the response bias decreased substantially from *b*_*ε*_ = +18.2 deg to *b*_ε_ = -2.3 deg. This result shows that the response changes in elevation were not confined to the six trained target locations on the midsagittal plane, but generalised across the two-dimensional frontal space (ruling out an explanation by speaker-specific peculiarities).

Interestingly, however, the LP6 and BS25 stimuli showed similarly strong improvements in localisation accuracy and precision for this subject. For the LP6 stimuli, which were the sounds with strongest deterioration of spectral-cue information, the regression values changed significantly (p < 0.001) from pre-training: [*g*_*ε*_, *b*_*ε*_, *r*^2^] = [0.2, 31.6^o^, 0.20], to post-training: [*g*_ε_, *b*_ε_, *r*^2^] = [0.4, 0.6°, 0.56]. For the BS25 sounds these changes amounted to, pre: [*g*_ε_, *b*_*ε*_, *r*^2^] = [0.3, 21.0°, 0.53], vs. post: [*g*_*ε*_, *b*_*ε*_, *r*^2^] = [0.7, 1.2^o^, 0.79].

**Figure 6:**
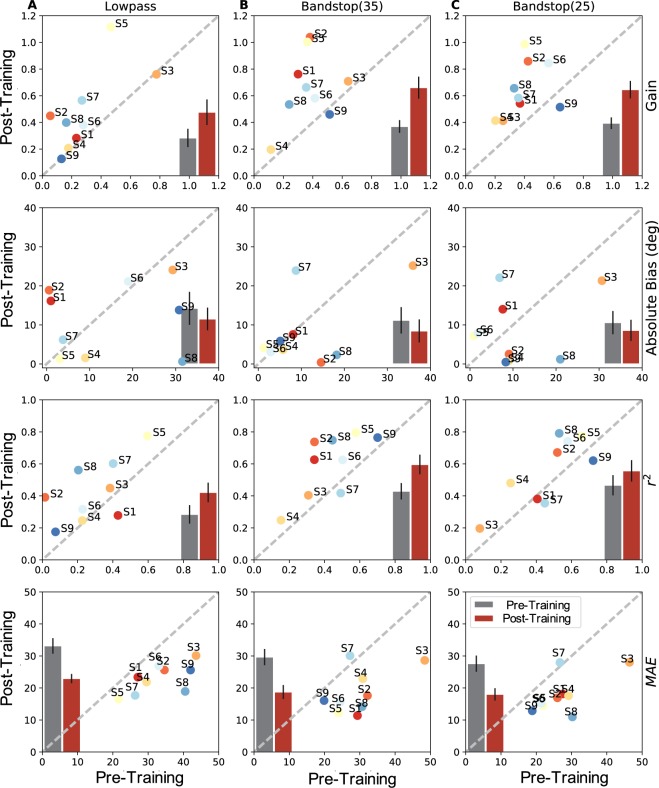
Summary of the regression analyses for all subjects shown as post-training vs. pre- training results. Columns: test stimuli; rows, from top to bottom: response gain, absolute response bias, coefficient of determination, mean absolute error. Averages across listeners for the two conditions are shown as insets: gray = pre-training with SEM, red = post-training with SEM. For the three stimuli, the post-training results were more accurate and precise.

Figure 6 shows the compared regression results for the LP6 (left-hand column), BS35 (center) and BS25 (right) stimuli between the pre-training (abscissa) and post-training (ordinate) experiments for all individual subjects (identified by the coloured symbols), together with the means and standard error of the means (gray bars for pre, and red bars for post, respectively) for the different regression parameters (from top to bottom: gain, absolute bias, r^2^, and MAE).

If the training would not have led to improved localisation, the data points should scatter evenly along the main diagonal, and the bars for the pre- and post-data would be indistinguishable. For an improvement, the gain and *r*^*2*^ data should lie above the diagonal, whereas the MAE and absolute bias data should lie below the diagonal. Although the bias results scatter more widely across subjects, showing no overall significant effect for the population, the other three regression parameters demonstrate clear improvement of localisation performance for all three stimulus types.

Table 2 provides the result of a one-sided sign test on these data, for the elevation response components to the three stimulus types, indicating that elevation localisation performance significantly improved for all three stimuli. This conclusion is further strengthened when also the small, but consistent, effects on the head-saccade reaction times are included. A shorter reaction time may suggest a higher level of confidence concerning the target location. The same 7/9 subjects showed slightly faster mean reaction times for the post-test localisation responses of all three stimulus spectra. Note that the post-training data were obtained late in the recording session, hundreds of trials after the pre-training data, when fatigue and reduced attentiveness may have started to affect reaction times (Fig. S3 in SI). As expected, no significant changes of response accuracy and precision were obtained for the azimuth response components (p = 0.08; see SI, Table S1, and Figs S4–S6, for details).

**Table 2:** One-sided sign test between pre- and post-training results.

Component	LP6	BS35	BS25
Elevation	29/36 p = 1·10^−4^	29/36 p = 1·10^−4^	27/36 p = 1·10^−3^

Comparison of pre- and post-regression results. n/36: number of data points out of 36 recording sessions per stimulus type that indicate an improved response (higher gain, higher r^2^, lower bias, and lower MAE).

### Experiment 2: Repetitive exposure to LP6 sounds without visual feedback

In the second experiment we tested three more subjects (S10–S12) on their localization performance to LP6 stimuli, when during the exposure session no feedback was provided for the same six locations as in Experiment 1. In the pre- and post localisation tests, responses were measured to BB and LP6 targets, randomly interleaved across the frontal hemifield. The basic response patterns for the azimuth components and summary figures for all three subjects are provided in the Supplementary Information (S4 and S9).

**Figure 7:**
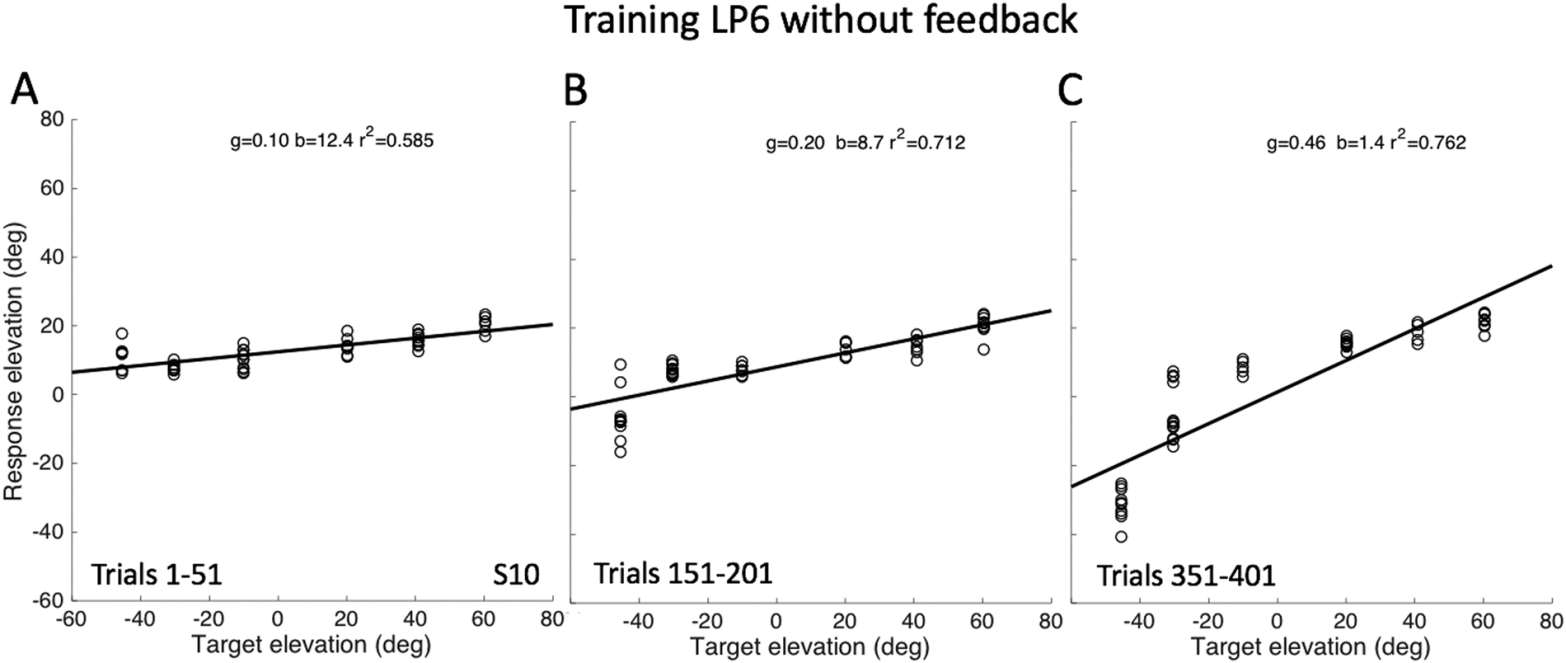
Localisation data for the six LP6 stimuli in the midsagittal plane, presented in randomized order without providing any feedback, (**A**) at the start (trials 1–51), (**B**) after 150 training trials (nrs 151–201), and (**C**) towards the end of the session (trials 351–401). Note the systematic increase of the response gain, and the reduction in response variability (increased *r*^*2*^) during the session. Note also that the response range expands most profoundly in the downward direction. Data from subject S10 (for the other two subjects, see Fig. S9 in SI).

Figure 7 shows that the response behavior of S10 gradually improved during the imprinting session for three selected blocks of 50 trials (similar format as Fig. 3). Both the response range and the response gain increased during the session, while the upward response bias and the coefficient of determination (variance) decreased during the open-loop exposure to LP6 stimuli.

**Figure 8:**
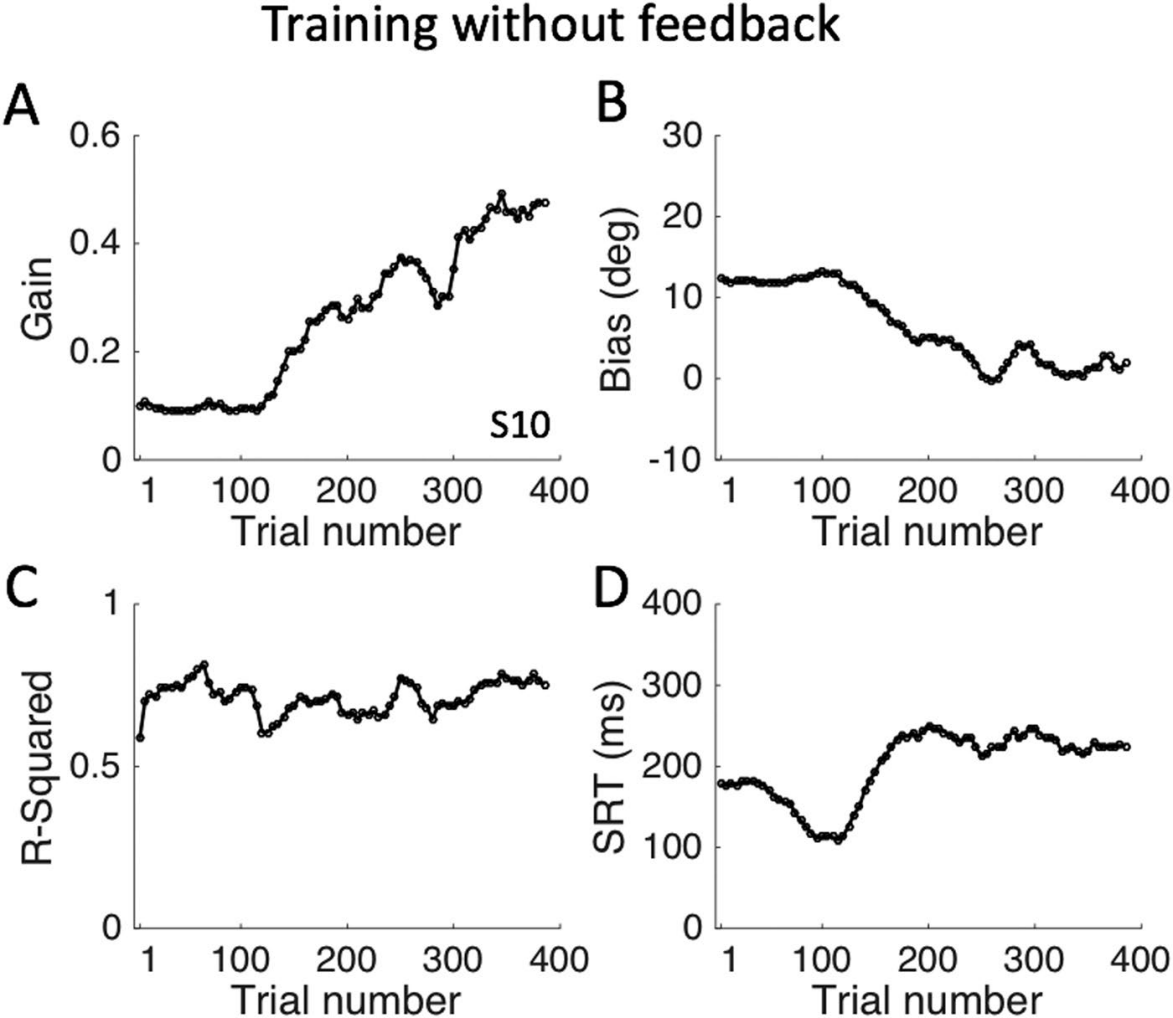
Localisation performance to the 432 trials of LP6 stimuli pseudo-randomly presented at one of only six locations on the midsagittal plane. (**A**) The response gain increases and (**B**) the bias gradually decreases during the open-loop training session. (**C**) The response variability stayed approximately constant during the block of trials. (**D**) Saccade reaction times initially decreased (as the gain remained constant, cf. with (**A**)), but during the adaptive phase they increased by about 125 ms. Regression coefficients were determined for blocks of 50 trials, moving in 5-trial steps along the exposure session of 432 trials. Data from subject S10 (for the other subjects, see Fig. S9 in SI).

To show how the response parameters varied during the entire open-loop exposure session, Fig. 8 shows response gain, bias, reaction time (SRT), and r^2^ as a function of trial number. Note that after about 120 trials the response gain systematically increased during the exposure session from about 0.1 to near 0.5, while at the same time the response bias decreased from about 12 deg to near zero deg. This means that, initially, the listener perceived all LP6 stimuli above the horizontal plane (bias about 12 deg; Fig. 7A), while during the sound exposure more and more downward percepts for stimuli presented below the horizontal plane emerged (Fig. 7B,C). The response reaction times initially started to decrease, but during the learning, they rapidly increased by about 150 ms. Meanwhile, the overall variability in the data (r^2^), remained roughly constant at about 0.7 (i.e., ~70% variance explained by the linear regression).

**Figure 9:**
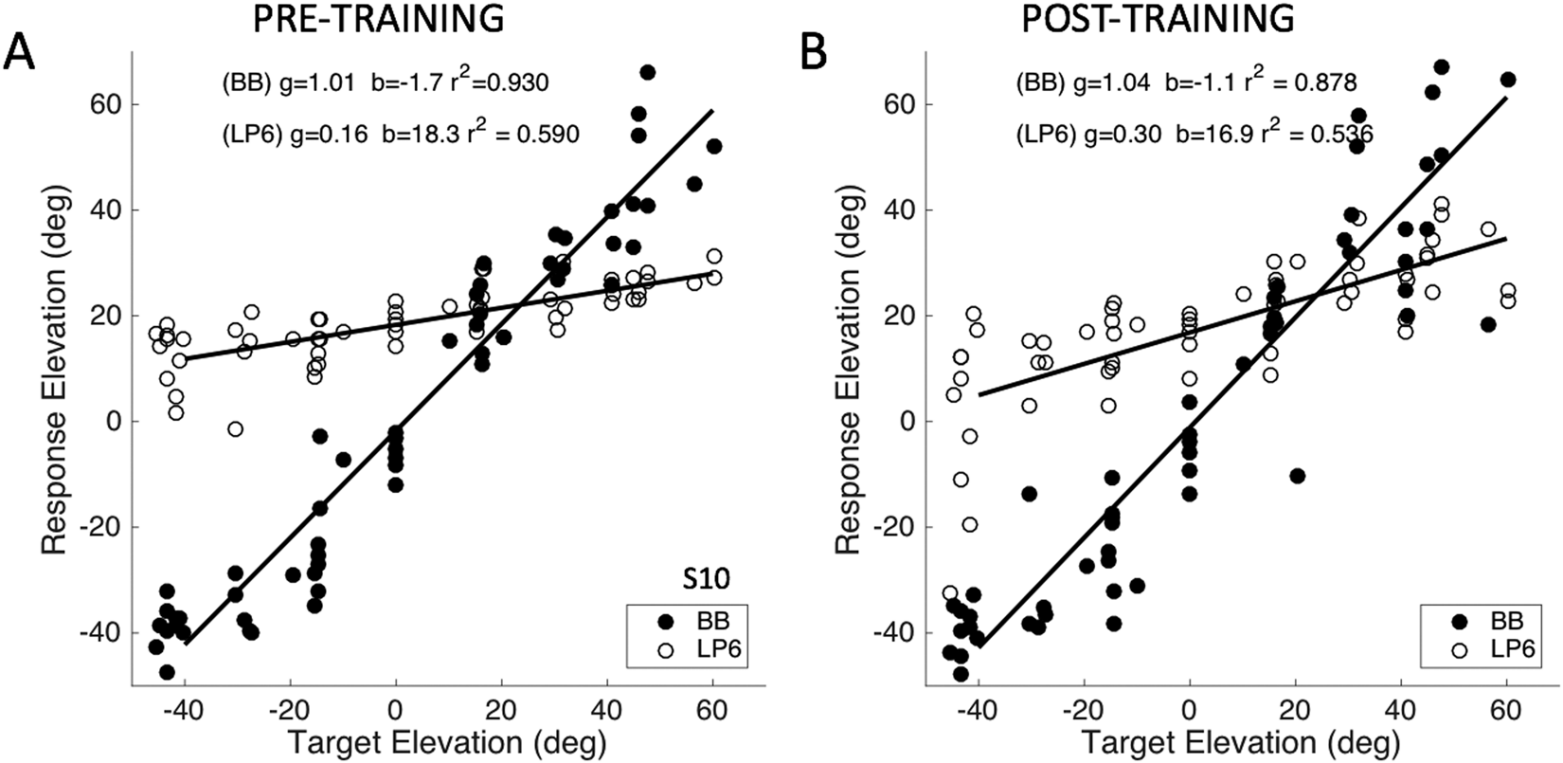
Stimulus-response elevation data before (**A**) and after (**B**) the open-loop training of localization to LP6 sounds. Filled symbols: responses to broadband sounds; open: LP6 sounds. After training, the LP6 responses were slightly better than before the training, which is evidence for transfer of the adaptation to the 2D frontal hemifield. Data from subject S10 (for the other subjects, see Fig. S9 in SI).

Figure 9 compares the pre- and post-training stimulus-response data in elevation for listener S10. Note that the responses to the BB stimuli were unaffected by the training. The Supplemental Material (Fig. S8) shows that the responses for the azimuth components of both stimulus types did not change either. The post-training responses to the LP6 sounds, however, were better (they had a higher gain, and a lower response bias) than before the training, although the effect was markedly smaller than for Experiment 1, where visual feedback was provided in each exposure trial.

## Discussion

### Perceptual learning

The results of our experiments demonstrate that the human auditory system can rapidly adjust its sound-localisation behaviour in the median plane on the basis of error feedback, or through repeated exposure to limited, yet spatially consistent, spectral cues, to improve its performance. Visual feedback, or open-loop sound presentation restricted to only a limited (n = 6) number of source locations, and for a single spectrally-impoverished stimulus (either BS35, or LP6), sufficed to induce changes in elevation performance that generalised to other target locations across the frontal hemifield, and for other spectral stimuli. Thus, rather than having learned a particular’categorisation strategy’ during the exposure or training phase, which would have enabled subjects to correctly identify each of the six speakers on the basis of particular acoustic characteristics in the sounds (unrelated to localisation per se), we argue that the auditory system implemented changes in its spectral-to-spatial mapping stage to improve its overall localisation performance.

It has been demonstrated before that perceptual learning can sometimes be induced without the use of any feedback or perceptible changes in the acoustic input^32^. Indeed, when performance error is not a driving force for changes in the percept or behaviour, the mere repeated exposure to stimuli could in principle lead to an improved detection or discrimination ability (‘perceptual learning’^32^). This could happen, for example, when being exposed repeatedly to a small, nearly imperceptible, change in an acoustic feature, like an amplitude modulation, a small frequency difference, or to a signal that is hidden in background noise. Our experiments show that also in the case of spatial hearing an explicit spatial error-feedback signal may not be always needed to improve source-localisation behaviour (Figs 7–9), albeit that the combination of repetitive stimulus exposure with visual feedback (Experiment 1) did provide a stronger and more reliable response improvement (Figs 2–5).

Clearly, subjects already must have had a weak notion about the subtle elevation-related cues in the low-frequency bands of the spectrum, which is evidenced by their nonzero response gains (Figs 2 and 7) in the control conditions. Repeated exposure to these weak cues may therefore have prompted the auditory system to gradually boost their importance for localisation. This mechanism would be further reinforced by explicit spatial (visual) error feedback, leading to an even stronger response enhancement. In the experiments, we ensured that stimuli (duration 150 ms) were too short to allow the head-orienting movements (with mean reaction times > 200–250 ms; Figs 8 and S3 in SI) to provide any valuable acoustic feedback through the systematic sensorimotor changes in the acoustic cues. We therefore conclude that the effects of the training, observed during the training phase (Figs 3 and 4) and after the training (Figs 5 and 6) were caused by visual error feedback in combination with the repeated exposure of impoverished, yet consistent, spectral cues (Figs 7–9).

We recently showed that manipulating the spectral contrast of the notch band (6–9 kHz) with respect to the surrounding lower and higher frequency bands systematically affected a listener’s elevation responses. The results suggested that the auditory system relies on the full acoustic spectrum to estimate the elevation angle^31^. A similar conclusion was drawn on the basis of response distributions to random spectral-shaped stimuli^15,29^. From the spectral-contrast results we hypothesized that the auditory system uses the weak elevation-related cues <5 kHz (Fig. 1) whenever they are available, but they contribute to the elevation percept with a lower weight than the much stronger cues within the 5–10 kHz notch-band. This spectral weight would thus express the reliability, or confidence, of the auditory system in the particular spectral cue to encode the elevation angle. The level of confidence eventually translates into the stimulus-response gain of goal-directed movements, when the sensory estimate is combined with internal priors about potential, expected source locations (see below).

Here, we further explored this idea, by providing repeated exposure with or without visual feedback to the listener for stimuli (BS35, LP6) that contained only impoverished information from the notch band. Note, however, that in contrast to the LP6 noises, the BS25 and BS35 stimuli also contained considerable acoustic power in the 9–20 kHz band. This high-frequency information is considered unreliable because of the poor hearing thresholds for normal-hearing humans, and the erratic (non-monotonic, rapidly varying) elevation cues at these high frequencies (e.g., Fig. 1). Yet, the data show that also these frequencies contributed to the elevation percept of our listeners, as the pre-training data and the control responses differed significantly for these stimuli when compared to the LP6 sounds (see Table 1 and Fig. 2).

The visual feedback helped listeners to gradually decrease their localisation errors across trials for the six BS35 stimuli, eventually leading to near-normal localisation behaviour for these sources (Fig. 4). Without the visual feedback, response accuracy increased as well, but never reached near-veridical performance (Fig. 8). The crucial test for the presence of true perceptual learning, however, is the generalisation of response behaviour to other stimuli than the ones that were imprinted during the training phase. The data show that response accuracy in elevation (quantified by the gain and bias of the stimulus-response relationships, and the mean absolute error) and precision (measured by the variability of the responses around the optimal regression line) had significantly improved after the training for all locations across the two-dimensional frontal space, as well as for the other two spectral test stimuli (LP6 and BS25). In terms of the hypothesis described above, we thus conjecture that the auditory system had increased the weights of the weak spectral elevation cues in the low-frequency band, to express its increased confidence in these cues for localisation. It is conceivable, that similar weight changes may have occurred in the high-frequency bands (9–20 kHz), but we have not tested this explicitly in our experiments with high-pass filtered noises.

### Neuro-computational model

Many studies that range from sensorimotor behaviour^26,32^, cognition^33,35^, perception^34,36^ to multi- sensory integration^37–39^ have consistently demonstrated that human behaviour, in the presence of sensory or motor uncertainty, can be understood from a statistical mechanism that relies on Bayesian inference.

**Figure 10:**
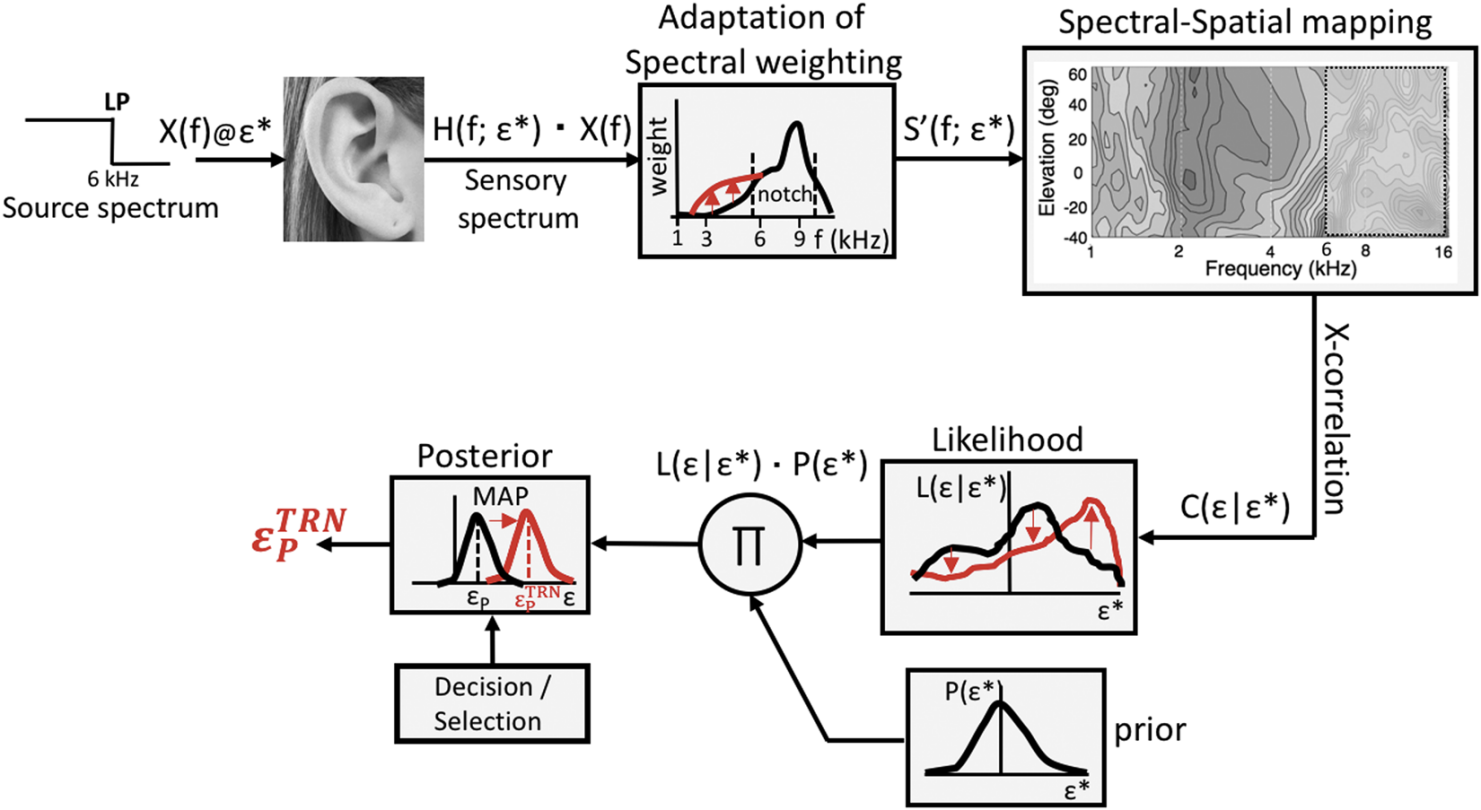
Conceptual model that explains how a limited input spectrum (LP) leads to an improved elevation percept after training. The sensory spectrum results from a convolution of the sound (here: X(f) = LP stimulus) with the HRTF of its elevation, H(f, ε^∗^). Frequency bands are subsequently weighted according to their quality and reliability of spatial information content. As a result of training, the weighting function is updated, by increasing the weights of low-frequency spectral cues (red). The resulting weighted sensory spectrum, S^′^(f, ε^∗^), is cross-correlated with stored information about all HRTFs (here: up to 6 kHz, as defined by the input), yielding a likelihood function of potential source locations, after rectification, L(ε; ε^∗^). Training shifts the likelihood from its initial estimate (black) to the learned estimate (red). Bayesian inference then weighs the (updated) sensory evidence against its prior (distributed around straight ahead), resulting in a changed posterior distribution. The maximum of the posterior (MAP) specifies the trained (red) perceived elevation angle, ε^TRN^, of the LP sound.

The neuro-computational model shown in Fig. 10 is an extended version of our earlier proposal on the spectral-to-spatial mapping stage in the human auditory system that could explain a veridical percept of source elevation, despite the ill-posed nature of the problem described in the Introduction^13,15,29,31^. In short, that model proposed that the auditory system contains learned representations of the full set of spectral HRTFs, and that the incoming sensory spectrum is cross- correlated with its internally stored data set over the relevant frequency range (e.g., between 3.5–14 kHz, depending on ear-size and hearing range). The result of this cross-correlation is a function of target elevation. It can be mathematically shown that if two prior assumptions are met: (i) all HRTFs are unique (i.e., they do not positively correlate with each other), and (ii) natural sound spectra do not resemble any of the stored HRTFs, that the maximum of the cross-correlation function will always point at the veridical elevation angle^13^. This strategy would allow the auditory system to successfully cope with a wide range of sounds, without having to assume that the source has a flat^3^, or sufficiently flat^6,40^, spectrum.

However, the examples in Fig. 1 suggest that the spectral cues in the HRTFs may have different information content: up to about 3 kHz, the cues do not seem to vary with elevation at all, but instead show a strong direction-independent amplification from the primary resonance in the ear canal (at about 2.5 kHz). From about 3.5 to 5 kHz the HRTFs start to diverge systematically (where they start to diverge is determined by pinna size^4,30^), albeit only slightly, thus providing a weak but systematic signal for source elevation, predominantly for downward elevation angles. These downward elevation cues may be the reason that our subjects started to expand their spatial percepts of the LP6 stimuli predominantly in the downward direction in the exposure without feedback (e.g. Fig. 8, and Supplemental Information S9), and much less to upward locations, as there are hardly low-frequency spectral cues for these upward elevations (e.g., Fig. 1). This weak signal, however, is vulnerable to interference, e.g. from background noise under normal environmental listening. We therefore hypothesised that the auditory system may consider the spectral cues in this frequency band as significant, but assigning them a relatively low weight to reflect their poor reliability and limited range. The same would hold for the very high frequencies (above ∼ 10–12 kHz), to which the human auditory system is relatively insensitive. In contrast, the cues within the notch band (5–10 kHz) are very prominent and systematic, cover the entire elevation space with sufficient resolution, and would contribute most reliably to estimate the true elevation angle of the target sound. As a result, their weights would be strongest.

In our extended model (Fig. 10), we included a spectral weighting function, w(f), that acts on the original sensory spectrum, prior to the cross-correlation stage^31^. The function reflects the system’s confidence in the different frequency bands to encode the elevation angle. It is assumed to be non-monotonic, and to peak in the central notch band. The result is a weighted sensory spectrum, S′(f; ε^∗^), which is determined by:

1 $${\rm{S}}^{\prime} ({\rm{f}};{{\rm{\varepsilon }}}^{\ast })={\rm{w}}({\rm{f}})\cdot ({\rm{H}}({\rm{f}};{{\rm{\varepsilon }}}^{\ast })+{\rm{X}}({\rm{f}}))$$

with X(f) the log-sound spectrum, and H(f;ε^∗^) the log-HRTF corresponding to target elevation, ε^∗^. The weighted sensory spectrum is subsequently cross-correlated with all stored HRTFs. This cross- correlation is performed only over the most relevant spectral bands in the signal (say, from 3.5–14 kHz). It yields a function of ε^∗^, C(ε|ε^∗^), which may contain peaks at those elevations (i.e., HRTFs) that most resemble the weighted sensory spectrum of Eq. 1. The likelihood function, L(ε|ε^∗^) is obtained from rectifying the correlation function, as only positive correlations may potentially relate to the true stimulus location.

A second extension to the cross-correlation model^13^ includes a stage that combines the likelihood function with an internal prior about expected source locations, P(ε^∗^) (here we assume a Gaussian distribution with a mean at zero elevation, and a fixed standard deviation, σ_prior_). Through Bayes’ rule, the multiplicative combination of likelihood and prior yields a posterior distribution that describes the probability of all possible source elevations giving rise to the sensory likelihood function:

2 $${\rm{POST}}({{\rm{\varepsilon }}}^{\ast }|{\rm{\varepsilon }})={\rm{L}}({\rm{\varepsilon }}|{{\rm{\varepsilon }}}^{\ast })\cdot {\rm{P}}({{\rm{\varepsilon }}}^{\ast })$$

Finally, the system’s elevation estimate is selected by a decision mechanism on this posterior distribution. For an optimal estimate (i.e., a minimum mean absolute error at minimum variance), this would be the Maximum A-Posteriori decision, or MAP, rule. It can be readily shown that the gain and variance of the elevation estimates from the MAP decision model depend on the noise (uncertainty) in the sensory input (i.e., the width of the likelihood function, σ_*ε*_), according to:

3 $${g}_{RESP}({\sigma }_{\varepsilon })=\frac{1}{1+\frac{{\sigma }_{\varepsilon }^{2}}{{\sigma }_{P}^{2}}}\,{\rm{and}}\,{\sigma }_{RESP}^{2}({\sigma }_{\varepsilon })=\frac{{\sigma }_{\varepsilon }^{2}}{{(1+\frac{{\sigma }_{\varepsilon }^{2}}{{\sigma }_{P}^{2}})}^{2}}$$

In the absence of sensory noise (σ_ε_ ≈ 0, which occurs for broadband GWN, or for the binaural extraction of source azimuth, for which σ_α_ ≈ 0; see Supplemental Information) the Bayesian decision stage yields a response gain close to one, and a variance that approaches zero. On the other hand, if the sensory noise is comparable to the uncertainty in the prior distribution, the response gain will be considerably smaller than one. This happens, e.g. for the sounds with weak spectral information content, like the LP6 stimuli.

We here conjecture that our perceptual learning paradigm induced reversible changes in the spectral weighting stage of the auditory pathway. The visual feedback, in combination with the repeated exposure to the same spectra and the system’s own orienting response, provided consistent sensory information regarding the subject’s elevation estimate, leading to an increase in the weights of the low end of the sensory spectrum as the localisation error decreased. Due to the increased contribution of these frequencies to the weighted sensory spectrum, the elevation estimate obtains an increased gain, and less response variance, by virtue of Eqn 3. More importantly, the changes in the spectral weighting function will not be confined to the particular stimulus for which the system was trained. As a result, also other sounds with similar impoverished spectral content, and presented at other locations in the environment, will yield improved localisation responses.

Note that our model incorporates the entire relevant frequency spectrum to estimate source elevation. Through the spectral weighting stage, it can thus allow for considerable flexibility, plasticity, and adaptive learning in response to acoustic, perceptual, or sensorimotor challenges. It is not trivial to apply these concepts to alternative models that rely fully on particular HRTF-specific spectral features (like steepness of a peak or notch in the spectrum, or presence of a covert peak) to estimate elevation^40–43^.

## Methods

### Participants

We report on the response behaviour of twelve listeners (Experiment 1: S1–S9, ages 22–30; 3 females, all naïve regarding the purpose of the experiments; Experiment 2: S10–S12, all male, and experienced in sound-localisation studies; two of the listeners were naïve as to the purpose of the experiment, one listener is author) who satisfied two inclusion criteria for the experiments: (i) normal-hearing sound-localisation performance to broadband Gaussian White Noise stimuli in azimuth and elevation, and (ii) a significantly degraded localisation performance in elevation for the band-limited sounds. Based on these criteria we excluded four other listeners, either because their localisation performance to GWN deviated substantially from normal-hearing performance (two listeners) or because their localisation performance for the BS35 stimuli (see below) did not deviate significantly from their GWN responses (another two listeners). The inexperienced subjects were given one or two brief practice sessions to get acquainted with the setup and localisation paradigms, and to gain stable localisation performance to standard broadband Gaussian white noise stimuli. All subjects reported to have no hearing problems of any kind, which was supported by their sound-localisation behaviour.

### Ethics statement

The local Ethics Committee of the Faculty of Social Sciences of the Radboud University approved the experimental procedures (protocol nr. ECSW2016-2208-41), as they concerned non-invasive observational experiments with healthy adult human subjects. All experiments adhered to the relevant guidelines and procedures for which ethical approval was obtained. Prior to their participation in the experiments, all subjects gave their full written informed consent.

### Experimental setup

During the experiments, the subject sat comfortably on a chair in the center of a completely dark, sound-attenuated room (length x width x height: 3 × 3 × 3 m). The floor, ceiling and walls were covered with sound-attenuating black foam, effectively eliminating echoes for frequencies >500 Hz. The room had an ambient background noise level of 30 dB A-weighted.

In Experiment 1, the chair was positioned at the center of a vertically-oriented circular hoop (1.2 m radius), on which 58 small broad-range loudspeakers were mounted (Visaton SC5.9), with a green LED in their center that could serve as a visual stimulus^24^.

In Experiment 2, the chair was at the center of a spherical acoustically transparent wire frame, on which 125 speakers were attached, spanning −120 to +120 deg in azimuth, and −55 to +90 deg in elevation (see Supplemental Information, Fig. S2).

Target locations and head-movement responses were transformed to double-pole azimuth-elevation coordinates^44^. In this system, azimuth, α, is defined as the angle between the sound source, the center of the head, and the midsagittal plane, and elevation, ε, is defined as the angle between the sound source, the center of the head, and the horizontal plane. The origin of the coordinate system corresponds to the straight-ahead speaker location. Note that for the total frontal hemifield in this system: |α| + |ε| ≤ 90° (see Fig. S2).

Head movements were recorded with the magnetic search-coil induction technique^45^. To that end, the participant wore a lightweight (150 g) helmet consisting of two perpendicular 4 cm wide straps that could be adjusted to fit around the participants head without interfering with the ears. On top of this helmet, a small coil was attached. From the left side of the helmet, a 40 cm long, thin aluminum rod protruded forward with a dim (0.15 Cd/m^2^) red LED attached to its end, which could be positioned in front of the listeners eyes and served as an eye-fixed head pointer for the perceived sound locations. Two orthogonal pairs of 2.45 × 2.45 m coils were attached to the edges of the room to generate the horizontal (60 kHz) and vertical (80 kHz) oscillating magnetic fields. The head-coil signals were amplified and demodulated (Remmel Labs, Ashland, MA), before being passed to 150 Hz anti-aliasing low-pass filters (custom-made, 4th-order Butterworth), and subsequently stored on hard disk at a sampling rate of 500 Hz per channel for off-line analysis.

### Auditory Stimuli

Acoustic stimuli were digitally generated using Tucker-Davis Technologies (TDT) (Alachua, FL) System III hardware, with a TDT DA1 16-bit digital-to-analog converter (48828.125 Hz sampling rate). A TDT PA4 programmable attenuator controlled sound level, after which the stimuli were passed from the TDT HB6 buffer to one of the speakers in the experimental room. Absolute free-field sound levels were measured at the position of the listeners head with a calibrated sound amplifier and microphone (Brüel and Kjaer, Norcross, GA).

The speakers had a nearly flat response characteristic between 0.02–20 kHz: fluctuations in their amplitude characteristics remained within ±3dB between 200 and 3000 Hz, and within ±2dB across the high end of the spectrum >3 kHz, which were not corrected for in the stimulus generation (see Discussion).

For examples of transfer characteristics of these speakers, we refer the reader to the manufacturer’s website at http://www.visaton.com/en/industrie/breitband/sc5_9nd_8.html.

All acoustic stimuli were derived from a standard Gaussian white noise stimulus, which had 5 ms sine-squared onset and offset ramps. This broadband control stimulus had a flat amplitude characteristic between 0.2 and 20 kHz, random phase, and a duration of 150 ms. The auditory stimuli, used in the training and exposure experiments, differed from the GWN control in their spectral content within and outside the 6–9 kHz band (the notch band; see Fig. S1). Bandstop (BS) stimuli were created by systematically attenuating the intensity between 6–9 kHz by −35 dB (BS35 stimulus), −25 dB (BS25 stimulus), or −15 dB (BS15 stimulus). The low-pass (LP6) filtered stimuli only contained band-limited noise between 0.5 to 6 kHz. This latter stimulus was chosen as it provided little, yet consistent, information about the elevation direction (see Fig. 1).

### Experimental paradigms

#### Calibration

Each experimental session started with a calibration paradigm to establish the mapping parameters of the coil signals to known target locations. Head- position data for the calibration procedure were obtained by instructing the listener to make an accurate head movement while redirecting the dim LED in front of the eyes from the central fixation LED to each of 58 peripheral LEDs, which was illuminated as soon as the fixation point extinguished. The 58 fixation points and raw head-position signals thus obtained were used to train two three-layer neural networks (one for azimuth, one for elevation) that served to calibrate the head-position data, using the Bayesian regularization implementation of the back-propagation algorithm (MatLab; Neural Networks Toolbox) to avoid overfitting^46^.

In each sound-localisation experiment, the listener started a trial by fixating the central LED (azimuth and elevation both zero; Fig. S1). After a pseudo-random period between 1.52.0 sec, the fixation LED was extinguished, and an auditory stimulus was presented 400 msec later. The listener was asked to redirect the head by pointing the dim LED at the end of the aluminum rod to the perceived location of the sound stimulus as fast and as accurately as possible.

### Control session

The sound-localisation experiments were divided over two experimental days. The localisation control experiment was performed on the first day. This experiment contained 275 trails with broadband, low-pass and band-stop (at −35, −25 and −15 dB) stimuli, and were presented at randomly selected locations that ranged from [−20, +20] deg in azimuth, and from [−50, +60] deg in elevation (see Fig. S2 in Supplemental Information). The control experiment served to establish the subject’s localisation abilities, and to verify the effect of low-pass filtering, or spectral attenuation in the 6–9 kHz band, on their localisation performance, prior to the training experiment.

The pre-training, training, and post-training experiments were performed on a second recording day.

### Training sessions

Experiment 1: In the training experiment, subjects localised the BS35 stimuli, presented at 6 fixed locations in the elevation direction (+60, +40, +20, −10, −30, −50 deg), and azimuth zero. After the sound was presented, and the subject had made the localisation response, a green LED in the center of the speaker was illuminated for a duration of 1500 ms. The subject was required to make a subsequent head-orienting response to the location of the LED; this procedure ensured that the subject had access to signals related to programming a corrective response, immediately after a sound-localisation estimate. The training experiment consisted of 432 trials, in which every location was presented 72 times in pseudo-random order.

Experiment 2: In this experiment, the same number of training trials was applied, but subjects now responded with a head-orienting response to the LP6 stimuli (presented at the same locations as in Experiment 1), without obtaining any feedback about their performance.

### Test sessions

Experiment 1: The pre- and post-training experiments contained the same 135 trials with three types of stimuli: LP6, BS35, and BS25 sounds (45 trials per stimulus). Stimuli were presented at pseudo-randomly selected locations from the 2D frontal hemifield, ranging from [−90, +90] deg in azimuth, and from [−55, +85] deg in elevation (Fig. S2).

Experiment 2: The pre- and post-training experiments contained 116 trials with two types of stimuli: BB noise and the LP6 sound. Stimuli were presented at pseudo-randomly selected locations from the 2D frontal hemifield, ranging from [−50, +50] deg in azimuth, and from [−50, +70] deg in elevation.

### Data Analysis

A custom-written MatLab script was used to automatically detect saccades in the calibrated data by using preset velocity criteria (15 deg /s) to saccade onset and offset^13^. Detected saccades were visually inspected for errors and manually corrected if necessary, without having access to stimulus information.

We analyzed the responses for each participant, separately for the different stimulus types, by determining the optimal linear fits for the stimulus-response relationships for the azimuth and elevation components:

4 $${{\rm{R}}}_{{\rm{\alpha }}}={{\rm{g}}}_{{\rm{\alpha }}}\cdot {{\rm{T}}}_{{\rm{\alpha }}}+{{\rm{b}}}_{{\rm{\alpha }}}\,{\rm{and}}\,{{\rm{R}}}_{{\rm{\varepsilon }}}={{\rm{g}}}_{{\rm{\varepsilon }}}\cdot {{\rm{T}}}_{{\rm{\varepsilon }}}+{{\rm{b}}}_{{\rm{\varepsilon }}}$$

by minimizing the least-squares error using the Scikit-learn library^47^. R_α_ and R_ε_ are the azimuth and elevation response components, and T_α_ and T_ε_ are the azimuth and elevation target coordinates. Fit parameters, b_α_ and b_ε_, are the response biases (offsets; in degrees), whereas g_α_ and g_ε_ are the response gains (slopes, dimensionless) of the azimuth and elevation responses, respectively. Note that an ideal listener should yield gains of 1.0, and offsets of 0.0 degrees. We also calculated Pearsons linear correlation coefficient, r, the coefficient of determination, *r*^2^, the mean absolute residual error (MARE), and the mean absolute localisation error (MAE) of the response, R, for each fit:

5 $$MAR{E}_{c}=\frac{1}{N}\sum _{n=1}^{N}|{R}_{c}-({g}_{c}\cdot {T}_{c}+{b}_{c})|\,{\rm{and}}\,MA{E}_{c}=\frac{1}{N}\sum _{n=1}^{N}|{R}_{c}-{T}_{c}|\,{\rm{with}}\,c=\alpha ,\varepsilon $$

### Statistics

To assess an effect of the training on the difference between the pre- and post-training results (i.e., gain, bias, r^2^, localisation error), we grouped the data for the three spectral stimuli in the test sessions across the nine subjects (i.e. 36 values, per stimulus type, and per test), and determined a distribution-free, one-sided sign test of the differences. In the sign test, each difference between post- and pre-adapatation value per subject is treated as the outcome of a binomial test with *p* = 0.5 for the probability of being either larger (gain, and r^2^), or smaller (absolute bias, and MAE) than zero. A p-value < 0.01 is obtained when at least 24/36 measurements indicate an effect.

## Electronic supplementary material

Supplemental Material
