# Supplementary material for: Learning to localise weakly-informative sound spectra with and without feedback
Source: Sci Rep. 2018 Dec 18;8:17933. doi: 10.1038/s41598-018-36422-z (PMC6298951; doi:10.1038/s41598-018-36422-z)
Supplement: Supplementary file 1 — Supplemental Material [file 41598_2018_36422_MOESM1_ESM.zip]

**Supplemental Information**

**Learning to localise weakly-informative sound spectra**

**with and without feedback**

**Bahram Zonooz1, Elahe Arani1 and A. John Van Opstal1**

1 Biophysics Department, Donders Center for Neuroscience, Radboud University,

Heyendaalseweg 135, 6525 AJ Nijmegen, The Netherlands.

**Supplemental results experiment 1:**

**BS35 repetitive exposure with visual feedback.**

**S1. Sound spectra.**


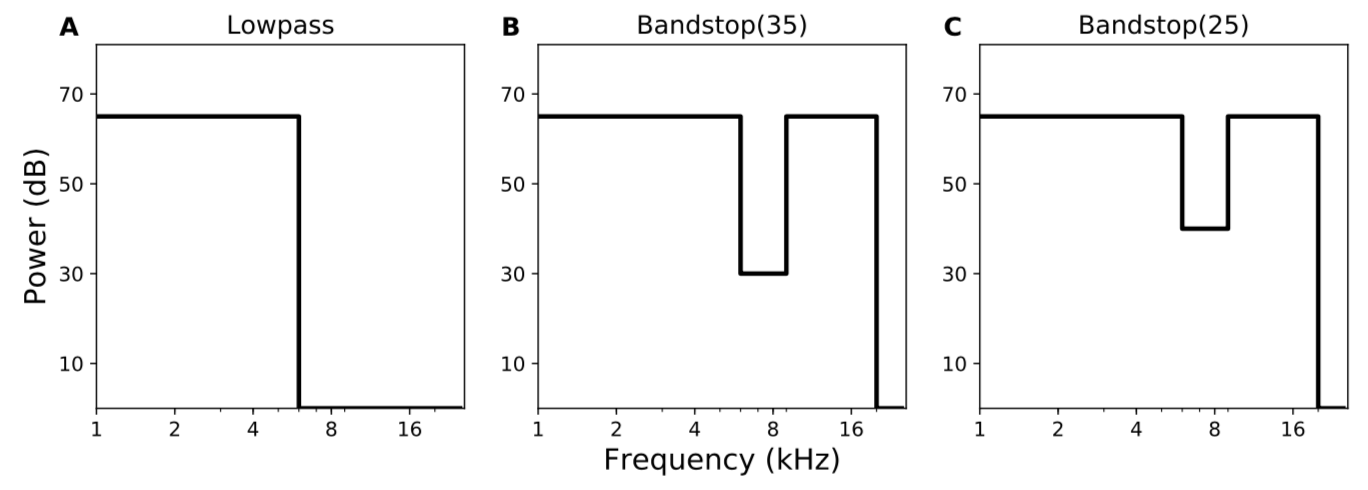


**Figure S1.** Schematised power spectra of the sound stimuli used in the training experiment. Stimuli were derived from a GWN control stimulus of 65 dB (A-weighted) by (A) removing all frequencies above 6 kHz (LP), or by attenuating the 6-9 kHz frequency band by (B) 35 dB, or (C) 25 dB.

**S2. Target distribution**


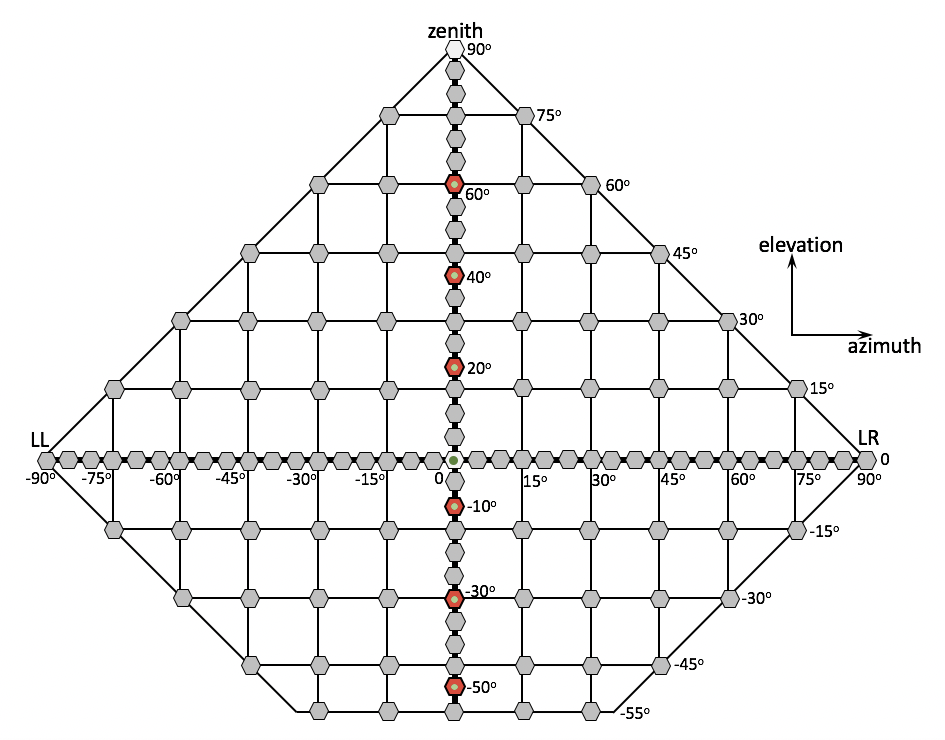


**Figure S2.** Distribution of sound-source locations, as used in the different experimental paradigms, projected onto a Cartesian azimuth-elevation coordinate grid. Note that speakers were attached to a spherical frame, and that in the double-pole azimuth-elevation coordinate system the sum of the azimuth and elevation angles can never exceed 90 deg (outer boundary of the plot). In the control experiment of day 1, the selected speaker locations were confined to [-20,+20] deg for azimuth, and [-50,+60] deg for elevation. The six training targets of the training experiment were located on the midsagittal meridian, and are indicated in red. They were presented with visual feedback (green dot) at the end of each localisation trial. The pre- and post-training test targets (red and dark gray) were distributed across the frontal hemifield, and were pseudo-randomly selected for azimuth in [-90,+90] deg, and for elevation in [-55,+85] deg. LL: lateral left, LR: lateral right. The central speaker at (0,0) deg, and the speaker at the zenith were not used.

**S3. Reaction-time dat**a

**Figure S3:** Influence of training on head-saccade reaction times. For the same 7/9 subjects the reaction times became slightly shorter after training. Only for subjects S5 and S8, responses were somewhat prolonged. Note that the post-training data were (necessarily) obtained after the 500 trials of the training phase, which could have slightly increased the post-training reaction times (due to fatigue), and therefore potentially underestimated the differences.

**S4. Azimuth results of the control experiment.**

| Stim | gain (std) | bias (std) (deg) | r2 (std) |
| --- | --- | --- | --- |
| GWN  BS15  BS25  BS35  LP6 | 1.1 ± 0.2  1.2 ± 0.2  1.3 ± 0.2  1.3 ± 0.2  1.3 ± 0.2 | 2±4  2±3  1±4  2±4  2±4 | 0.88 ± 0.06  0.90 ± 0.04  0.88 ± 0.07  0.90 ± 0.06  0.91 ± 0.03 |

**Table S1:** Azimuth results for the control experiment, averaged across subjects.

**S5. Azimuth stimulus-response relations: pre-training.**

**
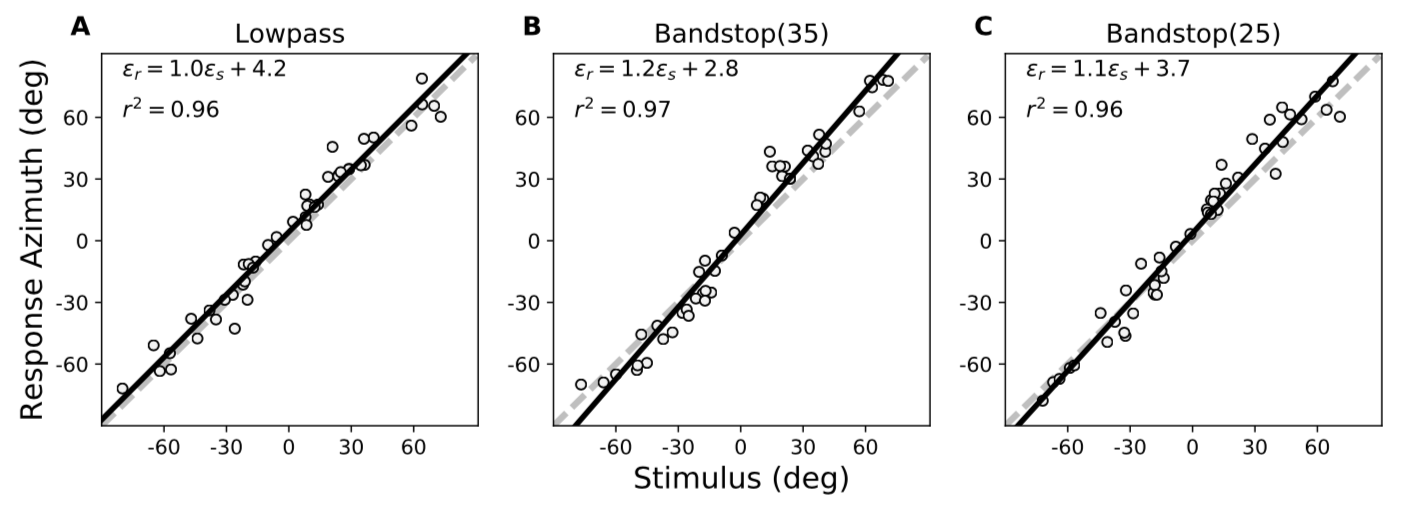
**

**Figure S4:** Pre-training results for subject S8 for the azimuth components of the three test stimuli. Dashed diagonals: perfect response behaviour. Note that responses were highly accurate for all three stimuli, as gains and biases were around their optimal values of 1.0 and 0.0 deg, respectively.

**S6. Azimuth stimulus-response relations: post-training**

**
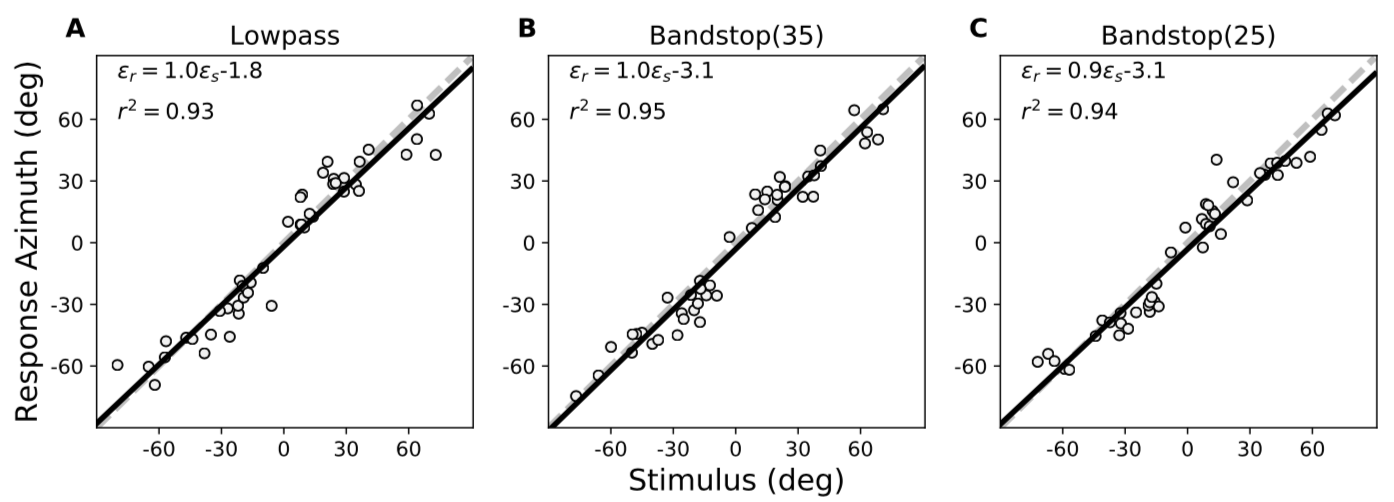
**

**Figure S5:** Post-training results for subject S8 in azimuth for the same three test stimuli as in Figure S4. Note absence of a change.

**S7. Azimuth data: comparison of pre- vs. post-training results**

**Figure S6:** Summary of the regression analyses of azimuth for all subjects shown as post- training vs. pre-training regression results. Data from were pooled for the three stimulus types, as localisation behaviour was independent of the spectral content (see Table S1). The pre- and post-training results for the azimuth response components were statistically indistinguishable. A binomial sign test on the 32 sessions yielded p = 0.08 (n.s.).

**Results of experiment 2:**

**Repetitive LP6 exposure without feedback.**

**S8. Azimuth response components pre- and post-exposure to LP6 sounds**

**
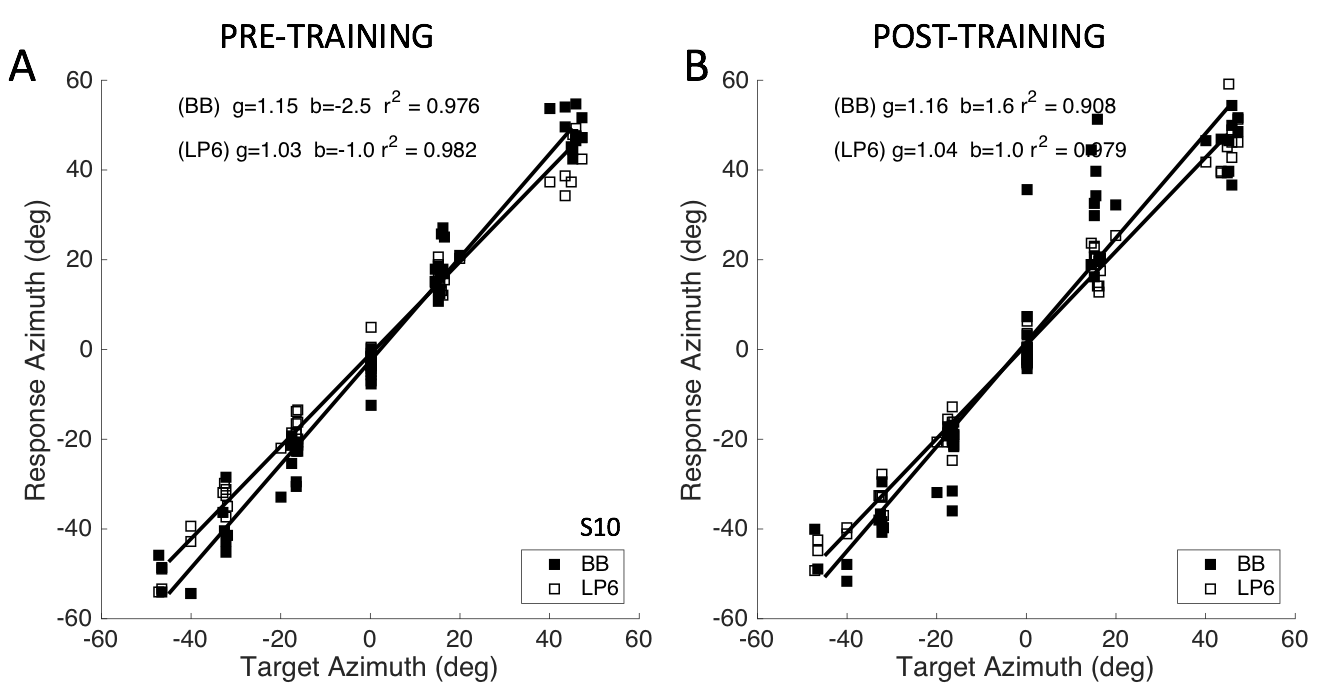
**

**Figure S7:** Azimuth response components of S10 to the BB and LP6 stimuli in the pre-training and post-training control experiments. Sounds were presented in the 2D frontal hemifield in Azimuth ([-50, +50] deg) and elevation ([-40, +60] deg), see also Fig. S2). The post-training results for the azimuth components were identical to the pre-training results for both stimulus types.

**S9. Elevation results of Experiment 2 for listeners S11 and S12.**


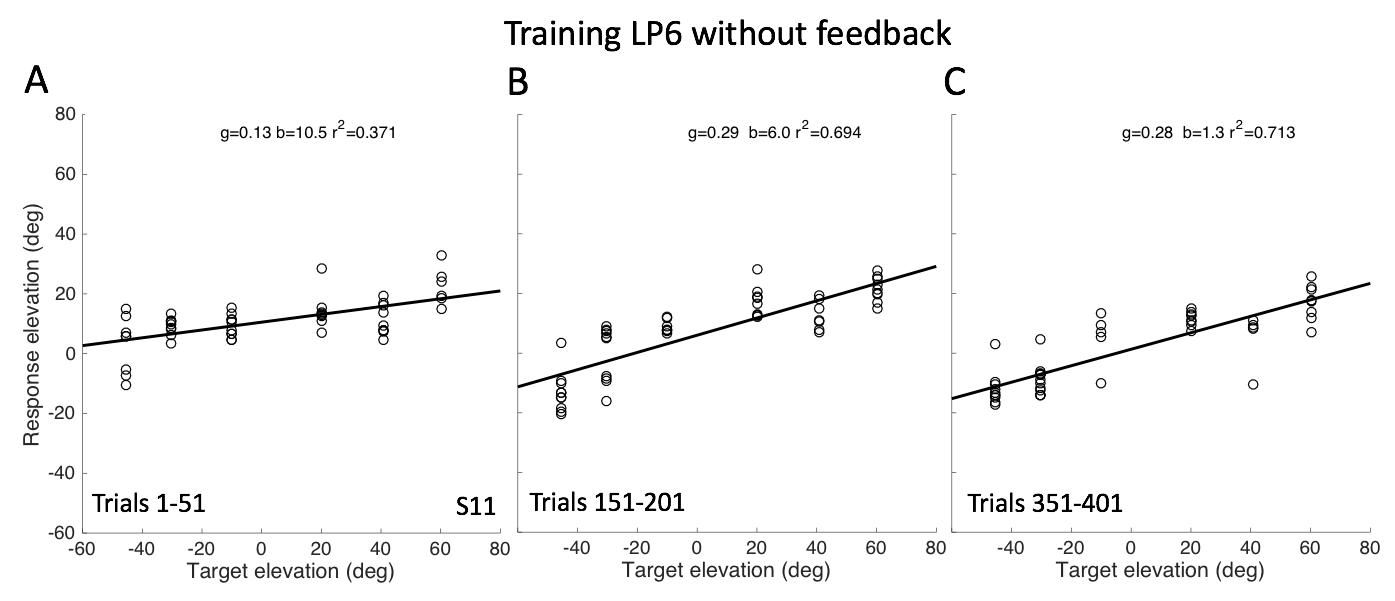


**Figure S8:** Regression on three different epochs during the LP6 exposure for subject S11. Same format as Fig. 7.


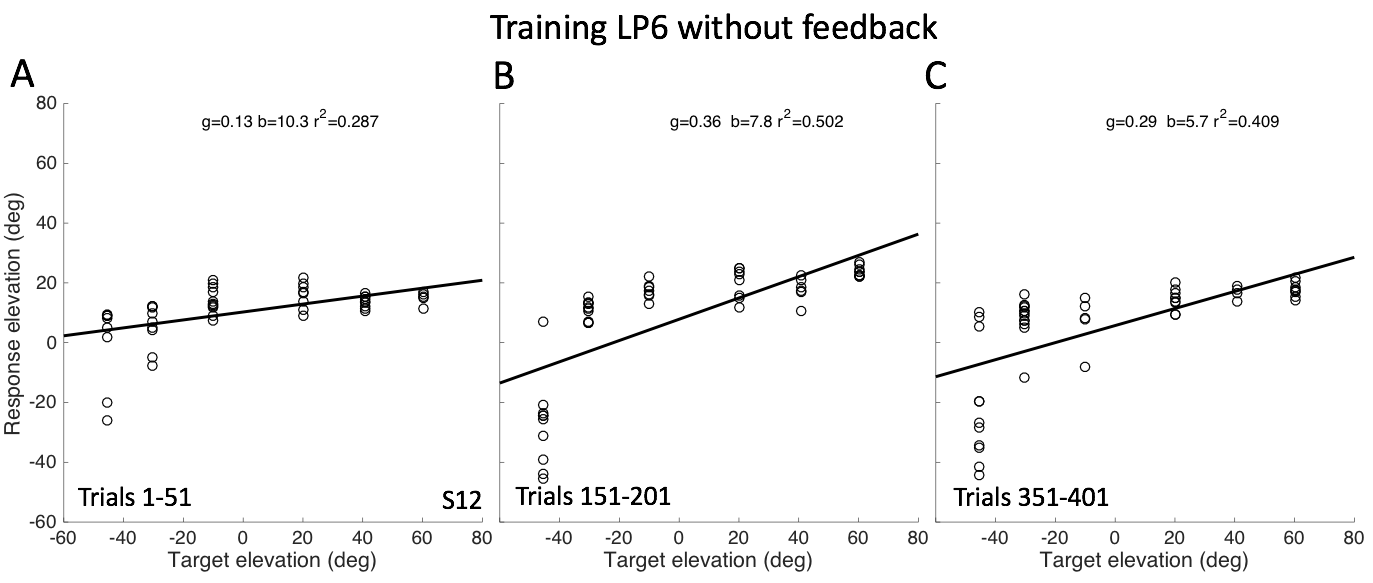


**Figure S9:** Regression on three different epochs during the LP6 exposure for subject S12. Same format as Fig. 7.


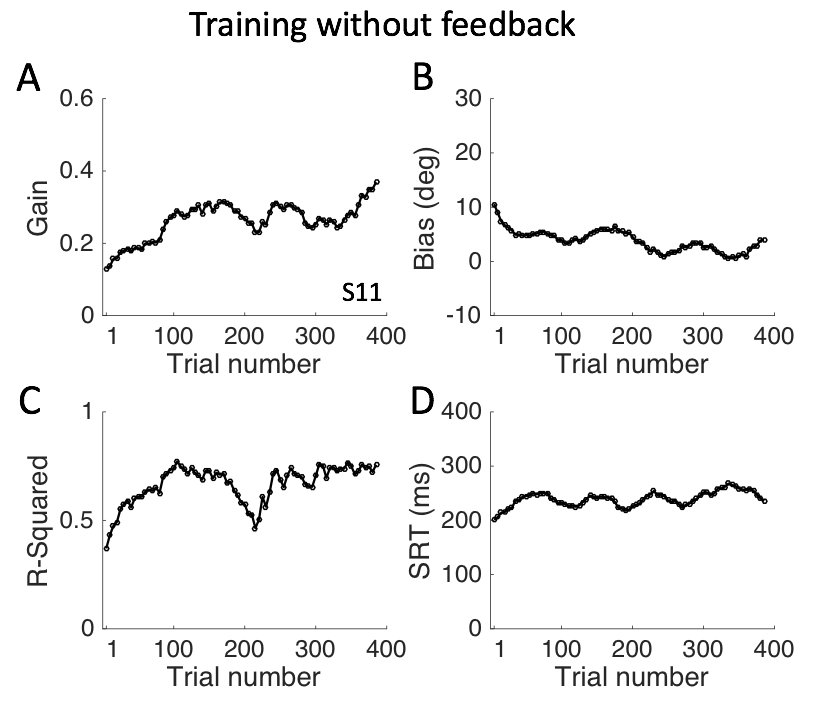


**Figure S10:** Results of repetitive exposure of LP6 sounds to 6 locations for S11. Same format as Fig. 8.


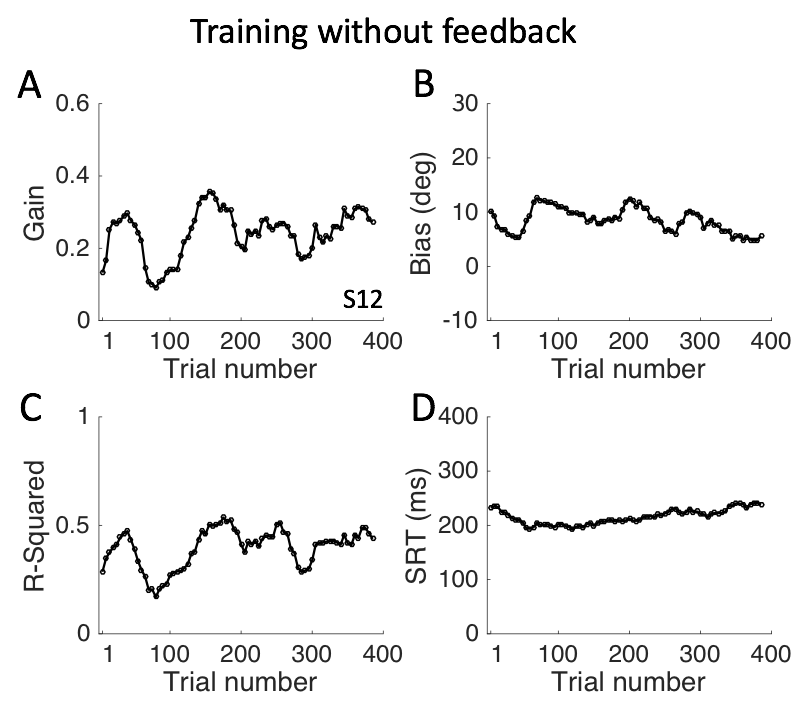


**Figure S11:** Results of repetitive exposure of LP6 sounds to 6 locations for S12. Same format as Fig. 8.


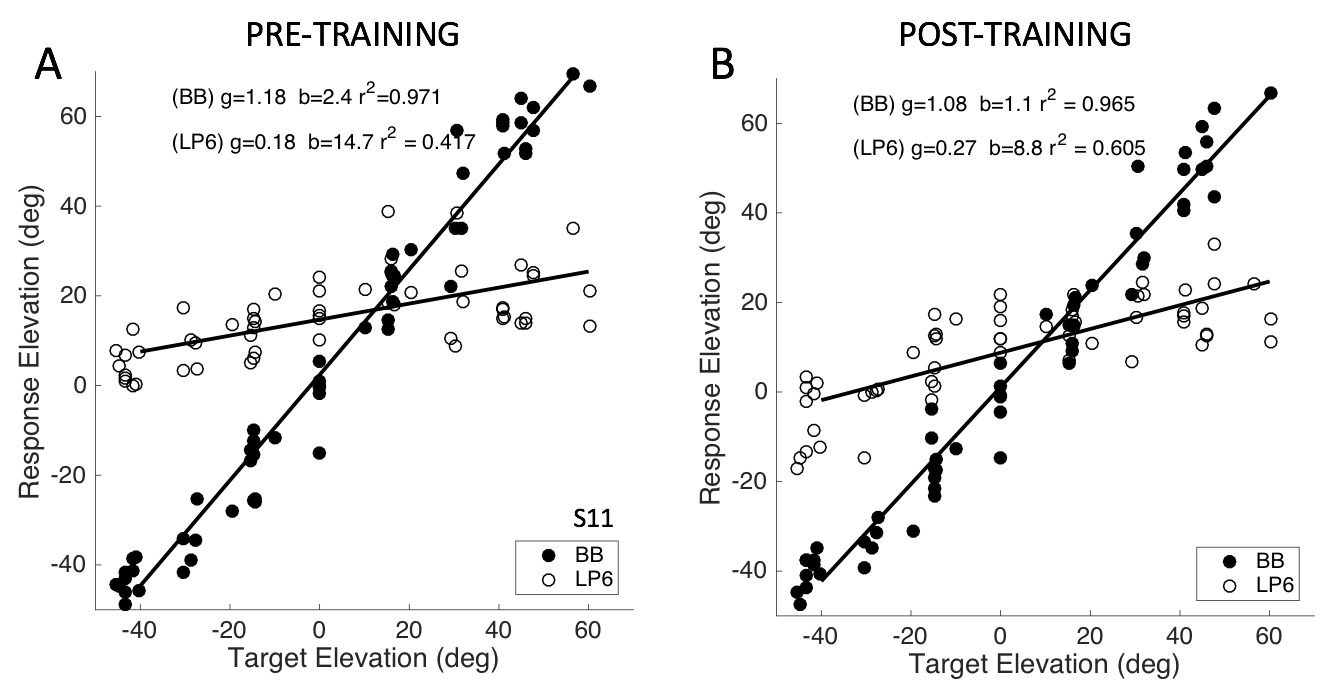


**Figure S11:** Regression results of the pre- and post-training data in elevation of Experiment 2 for listener S11. Same format as Fig. 9.


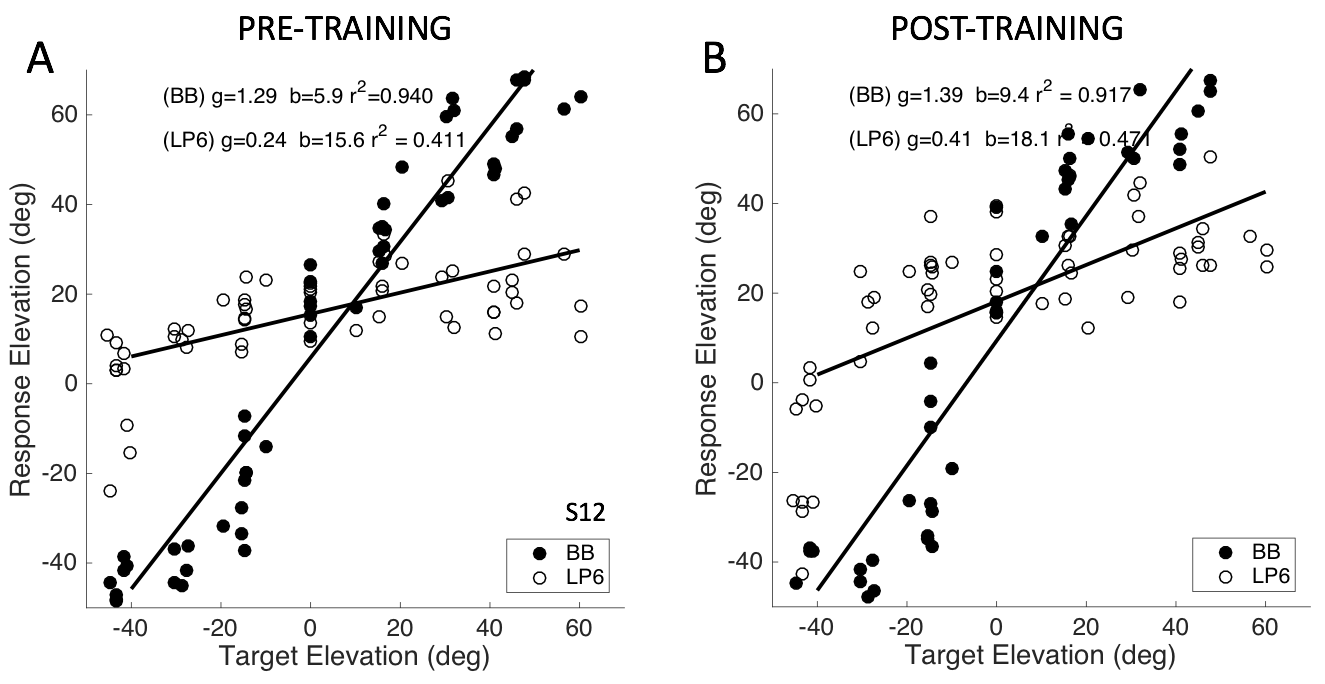


**Figure S12:** Regression results of the pre- and post-training data in elevation of Experiment 2 for listener S12. Same format as Fig. 9.
